# Current Bioinformatics Tools to Optimize CRISPR/Cas9 Experiments to Reduce Off-Target Effects

**DOI:** 10.3390/ijms24076261

**Published:** 2023-03-27

**Authors:** Muhammad Naeem, Omer S. Alkhnbashi

**Affiliations:** 1 Department of Bioengineering, King Fahd University of Petroleum and Minerals (KFUPM), Dhahran 31261, Saudi Arabia; 2 Information and Computer Science Department, King Fahd University of Petroleum and Minerals (KFUPM), Dhahran 31261, Saudi Arabia; 3 Interdisciplinary Research Center for Intelligent Secure Systems (IRC-ISS), King Fahd University of Petroleum and Minerals (KFUPM), Dhahran 31261, Saudi Arabia

**Keywords:** CRISPR/Cas9, bioinformatics, tools, sgRNA, deep learning, machine learning, off-target effects, base editors, prime editing

## Abstract

The CRISPR-Cas system has evolved into a cutting-edge technology that has transformed the field of biological sciences through precise genetic manipulation. CRISPR/Cas9 nuclease is evolving into a revolutionizing method to edit any gene of any species with desirable outcomes. The swift advancement of CRISPR-Cas technology is reflected in an ever-expanding ecosystem of bioinformatics tools designed to make CRISPR/Cas9 experiments easier. To assist researchers with efficient guide RNA designs with fewer off-target effects, nuclease target site selection, and experimental validation, bioinformaticians have built and developed a comprehensive set of tools. In this article, we will review the various computational tools available for the assessment of off-target effects, as well as the quantification of nuclease activity and specificity, including web-based search tools and experimental methods, and we will describe how these tools can be optimized for gene knock-out (KO) and gene knock-in (KI) for model organisms. We also discuss future directions in precision genome editing and its applications, as well as challenges in target selection, particularly in predicting off-target effects.

## 1. Introduction

**Figure 1:**
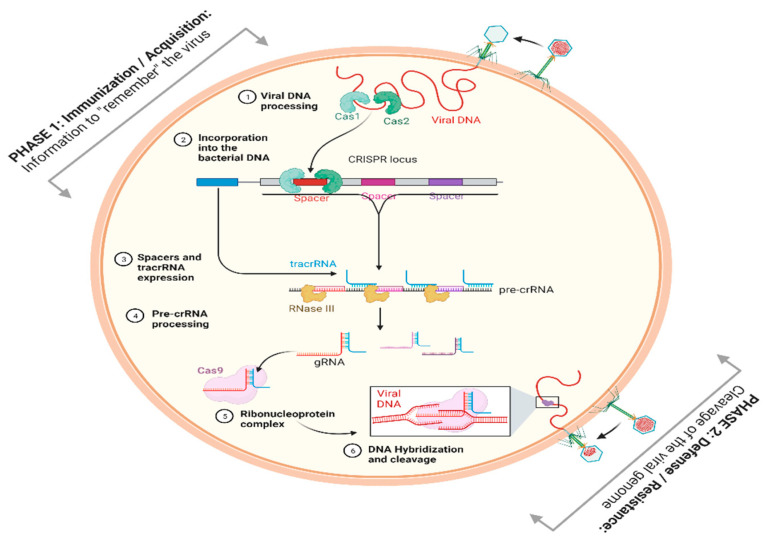
Schematic representation of CRISPR/Cas9 adaptive immune system. Adopted from Ghorbani et al. [9].

The unparalleled advancement in gene editing over the last decade has heralded a magnificent era in the field of biological sciences, especially genetic and genomic medicine. The impact of gene-editing technologies, particularly CRISPR/Cas, has been felt in nearly every field of biological sciences, including model organisms, biological evolution, agriculture, diagnostics, and therapeutics. Among the Clustered Regularly Interspaced Short Palindromic Repeats (CRISPR) CRISPR-associated proteins, Cas9 was discovered in bacteria and archaea as an adaptive immune system (Figure 1). Upon viral infection, it stores the viral information in pieces, which are called CRISPR arrays, defined as acquired genomic segments of viral DNA (spacer) separated by palindromic sequences. The transcription of these CRISPR-associated arrays leads to the formation of CRISPR-RNA (cr-RNA) or guide RNA. Cr-RNA combines with trans-activated RNA (tracrRNA), which is RNA that helps cr-RNA or gRNA bind to Cas9 to make the ribonucleic protein (RNP) complex. Upon viral infection, cr-RNA guides RNP complexes to cut (DSB) at complementary sites in the genome directly adjacent to a protospacer sequence motif (PAM), which is an NGG sequence in the case of Cas9. PAM sequences play a critical role in distinguishing between self- and non-self-DNA (viral DNA). Ultimately, Cas9 nuclease generates a double-strand break (DSB) three base pairs upstream of PAM sequences for gene knock-out (KO) or knock-in (KI) [1]. With the passage of time, the CRISPR/Cas9 system has been modified to broaden its application to solve more complex biological issues, such as cancer, cardiovascular disease, and emerging epidemics [2,3,4]. Due to its low cost, high efficiency, and high specificity, CRISPR/Cas9 has revolutionized both basic and applied sciences. In the laboratory setting, crRNA or gRNA contains only 20 nucleotides, which varies according to the targeted site in the genome. However, trans-activated RNA (tracrRNA) sequences remain constant when combining any Cas nuclease, such as Cas9, Cas12, etc., with cr-RNA or gRNA. Hence, there are two basic components of CRISPR/Cas9: gRNA and Cas9 nucleases [1]. As mentioned above, by alternating the 20 nucleotides of crRNA, the CRISPR/Cas9 system can be modulated to generate DSBs at any loci of the genome of any living organism. The DSB triggers two common types of DNA repair pathways, non-homologous end joining and the homologous direct repair pathway (HDR) based on the availability of template DNA. NHEJ leads to knock-out (KO) at the genomic level, and the HDR pathway leads to gene knock-in (KI) or the correction of faulty genes by providing single-strand oligo-DNA nucleotides (ssODNs) for point mutation or gene insertion [5,6]. The specificity of the CRISPR/Cas9 system depends on the complementary base pairing of gRNA with targeted sequences. However, partial sequence matching at any site of the genome between the gRNA and targeted genome leads to off-target cutting or off-target effects. These off-target effects generate lethal mutations in the exonic or intronic regions of genes or genomes [7]. In the last decade, after the discovery of CRISPR/Cas9, many computational tools have been created to design effective gRNA based on the prediction of on-target activity and off-target activity or effects. The computational tools used to facilitate the optimization of CRISPR/Cas9 experiments historically range from simple alignment-based approaches to calculating the scores of mismatches between the gRNA and target locus sequence and the incorporation of epigenetic data. Another class of tools has been created to evaluate the editing results through next-generation sequencing (NGS) or Sanger sequencing analyses [8].

In this critical review, we have comprehensively summarized the current knowledge of computational resources and key parameters for choosing the suitable computational tool for gene knock-out (KO), knock-in (KI), or knock-down (KD) experiments. We systematically discuss the various features of web-based tools for gRNA design and the quantification of off-target effects in vivo or in vitro from post-genome-editing data. All the discussed tools are extensively used in the research of animal and plant genome editing.

## 2. Role of Bioinformatics in the Development of CRISPR-Cas Technology

Bioinformatics played a key role in the discovery and development of CRISPR-Cas as a gene-editing technology. Firstly, in 1987, Yoshizumi Ishino identified some puzzling and mysterious DNA repeats with spacer genome sequences in bacteria [10]. However, he was unable to find these repeat functions due to the unavailability of bioinformatics dataset tools to analyze and find the similar/conserved domains of these mysterious repeats across genomic databases. After the invention of BLAST and the addition of the genome sequencing datasets for different species to NCBI, Francis Mojica discovered in 2005 that these mysterious repeats within the spacer, which were later called CRISPR arrays, do not belong to bacteria but are foreign genes [11]. After that, many bioinformatics analyses revealed that the spacer or CRISPR arrays belong to bacteriophages (viruses). This led to the development of the theory that the CRISPR-Cas system is an acquired immune system developed by bacteria over centuries to fight viruses [12,13]. Later on, Jennifer Doudna and Emmanuelle Charpentier (Nobel laureate 2020) modulated CRISPR-array-generated RNA (gRNA) to target the desired genomic sites in prokaryotic and eukaryotic cells to specifically edit their genomes, and this whole mechanism led to the development of the CRISPR/cas9 system as a precise RNA-guided gene-editing technology in the biological sciences [14]. Later on, bioinformatics tools were used to study matched spacer sequences across different genomes and find various types of Cas nuclease (Cas12, Cas12a, Cas10, etc.) to target RNA, DNA, and the latest proteins with high efficiency. Thus, bioinformatics played a very important role in the discovery and development of CRISPR/Cas9 technology.

Over the last two decades, bioinformatics tools have been developed in two tracks: (1) the analysis of coding genes and (2) the prediction of the interaction of nucleic acids at the genomic level. Shortly after the successful discovery and application of the CRISPR/Cas9 system in mammalian and field crop genomes, bioinformatics tools for purposes such as “to analyze the coding genes” were applied to classify different Cas variants through heuristic optimization tools and annotations. This classification led to the development of different types of CRISPR systems, such as 1V, V, and VI. Classification has been performed based on the structural component of cas genes and the architecture of CRISPR arrays [15,16]. Moreover, through the analysis of different archaeal, bacterial, and the latest viral genomes, the number of Cas gene families has increased to 27 and is further increasing day by day [17]. 

## 3. Optimized Workflow of CRISPR/Cas-System-Based Editing

**Figure 2:**
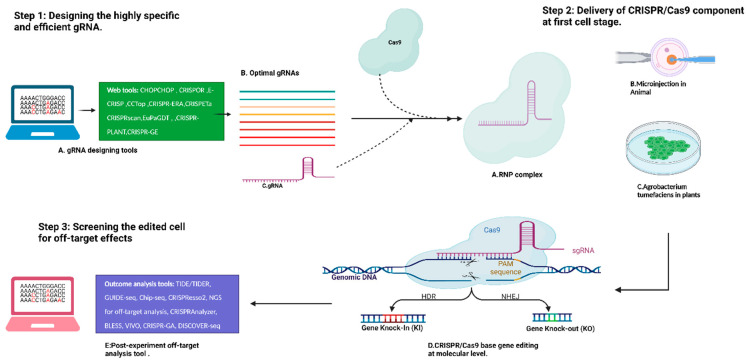
Schematic representation of three key steps of CRISPR/Cas9-based genome editing.

The main objective of CRISPR/Cas9-based genome editing is to modify the genes in genome sequences, which are further used to identify gene function, impair gene function, increase or decrease gene expression, and determine their potential applications in human therapeutics and crop genetic improvement [18,19]. To precisely modify the gene sequence in an optimized way, we need to follow three key steps (Figure 2). The choice of the key steps depends on the aim of the experiment: gene knock-out (KO), gene knock-in (KI), or transient or stable Cas9 expression. The universal key steps for CRISPR/Cas9 experiments are (1) designing highly specific and efficient gRNA, (2) delivering CRISPR/Cas9 components to the first cell stage, and (3) screening the edited cells for off-target analysis. These three steps are particularly crucial for any CRISPR/Cas9-based experiment. The first successful step for guide RNA design is very important for better and more successful outcomes. The ideal gRNA design has the two most important characteristics: (i) it should specifically complementarily bind to the target site at the genome level, and (ii) it should minimize other recognizable target sites or off-target effects [20]. For designing efficient and effective gRNA, many web-based user-friendly computational tools have been designed to identify the perfect gRNA with few or negligible off-target effects. In the second step, the delivery methods of CRISPR/Cas9 are very important for reducing off-target effects. There are many gene delivery approaches that have been designed to transfer the CRISPR/Cas9 components to plant and animal cells. For example, in plant cells, the *Agrobacterium tumefaciens* method has been established to transfer exogenous genes to targeted cells [21]. In the latest research, it has been identified that the delivery of the CRISPR/Cas9 system in the ribonuclease (RNP) form increases the on-target activity and significantly decreases the off-target effects. In the RNA process, a purified Cas9 protein combined with in vitro transcribed gRNA to form RNA complexes can be transferred directly to the first cell stage of the animal through microinjection (Figure 2) and to plant cells through electroporation [22]. After generating the DSB break, the Cas9 protein will be degraded, which leads to the transient expression of Cas9. This overall process has decreased the off-target effects [22]. Another factor is that different cells are biased toward DNA repair outcomes, and by modulating this bias, the off-target effects can be decreased [23].

There are many reviews available online in which authors have summarized the best techniques for transgenic development in animals and plants. After editing the targeted cells at the genomic level, it is highly necessary to screen the edited cells to assess the off-target effects and their intronic or exonic impact. Many computational high-throughput genome sequencing (NGS)-based in vivo and in vitro tools have been designed to analyze off-target effects after CRISPR/Cas9 experiments.

## 4. Off-Target Detection Methods: Biased and Unbiased

The CRISPR/Cas9 system in bacteria evolved as an adaptive immune system, protecting the bacterial cell from viral infections. The Cas9 targeting specificity is higher in the bacterial genome due to its smaller size as compared to mammalian genomes. Cas9 in bacteria and archaea evolved without selection pressure, so there is a high chance of off-target effects in the mammalian genome compared to bacteria and archaea [24]. The quantification of off-target effects and the assessment of their collateral genetic damage are effective ways to assess and develop reliable genome-engineering techniques as therapeutics. Since the discovery of CRISPR/Cas9, in the last decade, many algorithmic-based bioinformatics approaches have been created to detect and identify off-target effects to increase the on-target efficiency in any CRISPR/Cas9-based gene-editing experiment. All biased and unbiased tools have been described in our previous work [7]; now, in this review, we provide the latest updated list of biased and unbiased methods to detect off-target effects (Table 1).

### Latest Biased and Unbiased Off-Target Detection Methods

It is crucial for successful genome-editing experiments to design gRNA with minimal off-target effects. Initially, alignment-based models were developed, which simply aligned the gRNA sequence as an input and produced homology-based on/off-target effects as an output, much like the BLAST tool [25]. Later on, by employing regression and Elevation concepts, new tools were designed, but recently, with the application of deep learning and machine learning tools, the latest advanced off-target biased/unbiased detection web tools, such as MOFF, DeepCRISPR, PEM-Seq, GUIDE-Tag, and PEAC-Seq/TAPE-seq, have been generated for base and prime editing (Table 1). Together, on-target and off-target effects are assessed by DeepCRISPR. Recently, a novel model called MOFF that takes into account the two key elements that govern the mismatch tolerance, as well as the impact of epigenetics on the identification of off-target effects, was established. In tests based on CRISPR/Cas9, the recently developed MOFF model outperformed another model that was available to detect and quantify off-target effects [26,27]. Unbiased Primer-Extension-Mediated Sequencing (PEM-Seq) combined with LAM-HTGTS can be employed to detect and quantify off-target effects due to translocations at the genomic level with high sensitivity [28]. Based on the same strategy, another tool, GUIDE-Tag-Seq, has been designed to assess large deletions and translocation in CRISPR-edited genomes by tagging Tn5 augmentation to avoid bias in a simple PCR run. GUIDE-Tag is highly sensitive when it is employed to detect off-target effects where editing efficiencies are ≥0.2%. Regarding the CRISPR/Cas9 specificity assessment, see [29]. Compared to all unbiased in vitro and in vivo detection tools, GUIDE-seq is preferred due to its low cost, low false-positive rate, and application to diverse cell lines [30,31]. Recently, to identify off-target effects due to prime editing, researchers have also developed methods such as PEAC-seq and TAPE-seq. Prime Editor Assisted Off-target Characterization Sequencing (PEAC-Seq) has been employed to identify off-target effects and genotoxicity due to large translocations, both in vivo and in vitro, produced by prime editors [32]. Researchers recently developed a method to detect off-target effects in prime-edited cells, which is the TAPE-Seq genome-wide off-target detection method. TAPE-Seq can identify off-target effects in live cells. One benefit of TAPE-Seq is it can analyze both the on-target and off-target activities of prime editors [33].

**Table 1:** Latest biased/unbiased off-target detection methods.

Tools	Description Features	Cell Line	Study	Limitations	Ref.
DeepCRISPR	Deep learning tool to predict off/on-target hits together with DNA methylation factors	Human and mouse cell lines	In vitro/ in vivo	Not suitable for base editors and prime editors	[26]
MOFF	The latest multi-layer regression-based model to predict off-target effects by incorporating the GMT and new epigenetic factors along with other factors, such as sequence features, structure features, and epigenetic features	Human and mouse cell lines	In vitro/ in vivo	Specificity	[27]
PEM-Seq	Latest generated off-target detection method, which is highly sensitive in detecting genomic translocations in edited cells	Human and mouse lines	In vivo	Not suitable for base editors and prime editors	[28]
GUIDE-Tag	Latest in vivo developed method to detect off-target effects where editing efficiencies are ≥0.2%.	Mouse and human cell lines	In vivo	Cannot provide specificity information	[29]
PEAC-Seq	Unbiased method of off-target effect identification in the prime-edited cells.	Mouse and human cell lines	In vivo	Sensitivity	[32]
TAPE-Seq	In vivo method to detect both on- and off-target events generated by prime editors	Human cell lines	In vivo	Sensitivity	[33]

## 5. Efficient Guide RNA Design Tools

Efficiency and specificity are the two main core requirements to design the best possible guide RNA (gRNA). The term efficiency means how well the gRNA guides Cas9 to the target locus of any targeted genome or the percentage of cells that are edited. The term specifically demonstrates that CRISPR/Cas9-targeted editing sites are unique and common, leading to off-target effects [34]. Many factors affect the efficiency and specificity when designing guide RNA to target a specific locus with minimum off-target effects. These factors include sequence, structural, and epigenetic features (Table 2). CHOP CHOP was the earliest tool to design guide RNA based on a simple BLAST match or mismatch between gRNA and target sites in the genome of edited cells. CHOP CHOP is a diverse tool for designing gRNA for more than 100 organisms. The machine-learning-based off-target effect detection method Cas-OFFinder was generated and incorporated into the CRISPR RNGE web-based tool to design efficient gRNA by finding on-target and off-target effects [35,36]. CRISTA is a web-based tool to design gRNA based on the quantification of on-target and off-target effects. CRISTA can be employed to design effective gRNA for more than 100 organisms; the limitation of CRISTA is that it is supported only for spCas9. Based on the incorporation of sequence and structural features, GuideScan web-based tools were generated for designing gRNA specifically for editing the mouse and human genomes [35,37]. CRISPRDo is a web-based gRNA design tool specifically for Cas9 and cpf1 Cas nucleases. CRISPRDo can detect both on-target and off-target effects simultaneously. CRISPRDo can be specifically used for model organisms such as zebrafish, mice, humans, and some worm species [38]. sgRNACas9 is a web-based tool to quantify off-target effects based on certain structural and sequence features. sgRNACas9 is specifically used for the generation of spCas9 gRNAs to edit the mouse genome [39,40]. A recently developed web-based model for designing gRNA specifically to edit the genomes of eukaryotic pathogens is EupaDGT. EupaDGT is supported by various types of Cas variants [41]. WU-CRISPR is a machine-learning-based gRNAA designed only for spCas9 nuclease. WU-CRISPR is specifically used to design gRNA to edit the human and mouse genomes [42]. CRISPR-P is a web-based gRNA design tool usually employed for designing gRNAs to edit model plants’ and other field crops’ genomes. Recently, the web-based CRISPRz tool has been generated after training on a large database specifically for spCas9. Another web-based tool to design gRNA to edit plant genomes is PhytoCRISPR. PhytoCRISPR supports gRNA designs for spCas9 and Cas12 [43,44]. The widely used CRISPOR web-based tool is used to quantify off-target effects and then generate a series of gRNAs. CRISPOR can support more than 30 types of Cas variant nucleases. Recently, after the discovery of prime editing and base editors, a web-based tool (Png Designer) for designing base editor gRNA and prime gRNA has been designed [45,46,47].

### Properties to Consider for the Optimization of gRNA Design and Synthesis

To improve the guide RNA efficiency and specificity, in the last decade, different types of properties have been employed, such as sequence, structural, and epigenetic features, truncated gRNA, target site selection, and the Cas-to-gRNA ratio. Before designing gRNA, the PAM (protospacer adjacent motif, 5′NGG3′) sequence composition is important to ensure cleavage by Cas9. A single mismatch in PAM leads to off-target effects [48]. In one study, it was reported that, for the optimal gRNA design, “G” should be avoided in the first nucleotide position downstream of the extended PAM. The cleavage efficiency of gRNA decreases when gRNA contains poly-N motifs, five contagious A (such as AAAA) or C (CCCC) bases, etc. This proximal region is called the seed region, with a length ranging from 16 to 20 nts. Any mismatch in the seed region leads to off-target effects. In the seed sequence, U is disfavored. The most important nucleotide is at position 20, where G is strongly favored and C is disfavored. Adenine is favored at position 20, a purine (C, G) is favored at position 19, and C is favored at 18. At position 16, G is favored over other nucleotides. At position 17, the nucleotide preference is still unclear; some studies show a preference for G, whereas other studies show a preference for C and the depletion of G [49,50,51,52,53]. The RNA secondary structure is important for the site-specific cleavage of gRNA. The accessibility of certain nucleotides at position numbers 18 to 20 and 51 to 53 is important because these nucleotide positions are crucial for the scaffolding and formation of the secondary structure of the gRNA. If there are complementary sequences at 18 to 20 and 51 to 53, these lead to alterations in the secondary structure and decrease the accessibility of nucleotides and the efficiency of gRNA [54]. Some researchers applied thermodynamic principles to evaluate the overall stability of gRNA for efficient functions and design. Thermodynamically, the free energy of gRNA is an important feature for its intermolecular interaction with targeted nucleotides at the atomic level. In one study, it was reported that the high negative free energy of gRNA leads to compact folding and lower accessibility of gRNA to target sites. The thermostability of RNA is determined through the GC content percentage of gRNA nucleotides. The optimal GC content is more important than a higher or lower GC content. In a few studies, it has been reported that GC contents above 65% make the gRNA inefficient, and GC contents less than 40% make the gRNA inactive compared to the optimal GC content (40 to 60) [55,56]. In the epigenetic context, the chromatin structure is a major factor that impacts PAM identification by Cas9 and hence its ability to bind to the seed region of gRNA. For example, it has been observed that the N’-terminal site of the CD15 gene was an effective target locus due to the chromatin structural effect. As an epigenetic effect, the chromatic structural effect varies among cell types; for instance, the DNA CpG methylation assay can reflect an aspect of chromatin accessibility that is not fully captured by DNase hypersensitivity. Therefore, using multiple assays on various types of cells could better explain variations in sequence similarities. Following up on this issue, researchers have used multiple assays in repeated studies on various types of cells. They found that there is around a 20% chromatin structural effect on the efficiency of gene knock-out (KO) in mammalian cells [57,58,59]. Regarding truncated gRNA, it has been reported that the off-target effects decreased 500-fold when reducing the length of gRNA from 20 nucleotides to 17 nucleotides. Combining truncated gRNA with Cas nickase has significantly reduced off-target effects in mammalian cells. Dead RNA off-target suppression is a newly developed strategy in which mutated or dead gRNA guides Cas9 but suppresses the cleavage, decreasing the off-target effects and increasing the on-target activity [60,61]. In a recent study, it was reported that the gRNA and cas9 cleavage activity decreased when targeting sites close to the C-terminus. It has been suggested for knock-out (KO) designs that using gRNA near the first exon increases the chances of frameshift mutations. Designing gRNA on 5′ and 3′ UTRs also remains ineffective [62]. In many studies, it has been reported that increasing the concentration and ratio of gRNA and Cas nucleases increases the chances of off-target effects. In one study, it was shown that at the minimum ratio and concentration, there are high chances of on-target activity and fewer unintended mutations [63].

## 6. Bioinformatics Tools for Repair Outcome Predictions

CRISPR/Cas9 employs the NHEJ DNA repair pathway for gene knock-out and the homologous direct repair (HDR) pathway for gene knock-in. Recently, different types of computational tools (Table 3) have been generated to predict DSB repair by manipulating the biases of repair outcomes. The prediction of the DSB DNA repair pathway is crucial for designing the best possible CRISPR/Cas9 gene knock-out/knock-in wet lab experiment. Depending on the purpose of the CRISPR/Cas9 experiment, different types of DNA repair pathways are modulated. The DNA repair system in eukaryotic and prokaryotic cells is affected by multiple factors, such as the cell cycle stage and the secretion of repair-related proteins [64]. The most common DNA repair method for gene knock-out (KO) is canonical non-homologous end joining (C-NHEJ); recently, in some studies, this method has been altered to another method, microhomology-mediated end joining (MME), in some organisms. Both DNA repair methods lead to insertions and deletions (INDELS) [65]. In comparison to INDELS and MME, the HDR pathway, which occurs only in the G2 and S phases of the cell cycle, is precise and complex and repairs the DSB in a template-dependent manner [6]. In recent studies, it has been proved that the DNA repair outcome is not random; it can be specifically replicated, especially depending on the specific DNA sequences in the genome [23]. In recent studies, it has been validated that mutation outcomes vary from cell line to cell line. The accurate prediction of DNA repair outcome problems is difficult for bioinformaticians. Recently, machine-learning-based strategies have been employed to train and detect repair outcomes (Table 3).

## 7. Bioinformatics for Post-CRISPR-Experiment Off-Target Analysis

Various types of bioinformatics tools have been designed for the post-experiment analysis of off-target effects. Some widely used tools are TIDE, CRISPR-GA, Cas-Analyzer, and CRISPResso2 (Table 4). Tracking INDELS by decomposition (TIDE) is a Sanger sequencing-based tool to analyze all insertions and deletions that occur through CRISPR/Cas9. A new and advanced version of TIDE has been designed, Tracking Insertions, Deletions, and Recombination (TIDER), which can detect off-target effects introduced by both INDELS and HDR pathways [72,73]. Another group of researchers has employed NGS analysis for the post-experiment analysis of off-target effects; such tools are CRISPRpic and CrispRVarinat. These tools are based on the command line interface, which facilitates the detailed analysis of outcomes for naive and experienced users [74,75]. Since the creation of these tools, many more user-friendly tools to detect off-target effects after experiments have been generated, such as CRISPR-Genome Analyzer (CRISPR-GA). CRISPR-GA employs NGS data analysis to quantify INDELS and recombination through HRD repair pathways. The protocol pipeline of CRISPR-GA consists of quality control reads, which quantify the editing efficiency and occurrences of NHEJ and HDR repair pathways in the genome [76]. As another Javascript-based data analysis through the NGS tool, Cas-Analyzer has been generated. This program works like CRISPR-GA, but the one advantage over CRISPR-GA is that the working speed of Cas-Analyzer is faster than CRISPR-GA. Moreover, it supports the analysis of various types of Cas9 nucleases [77]. Another updated version of the CRISPResso tool is CRISPResso2. CRISPResso2 can help to quantify allele-specific off-target effects. This tool is useful to identify the off-target effects generated through newly designed base editors for specific substitution. The current major drawback of this tool is the limited upload data, 100 MB in a FASTQ file [78]. The latest developed method for post-experiment off-target analysis is Inference of CRISPR Edits (ICE), which detects off-target insertions and deletions through the employment of the Sanger sequencing method. ICE is a regression-based algorithm characterized by robustness and versatility that can be applied to quantify INDELs shortly after the transfer of CRISPR reagents to the targeted cells [79].

All of the above-mentioned tools can analyze the off-target and on-target sites in silico. All of the above approaches are biased toward Crispr/cas9 experiments. There are many limitations associated with biased techniques, such as unpredictable omissions or less likely cleavage, leading to off-target effects. The limitation of these tools can be overcome by applying the whole-genome sequencing method. However, the whole-genome sequencing technique is highly expensive and not suitable for the analysis of many clones of edited cells. Then, unbiased methods were developed through advanced machine learning and deep learning techniques, as mentioned in Section 2 (GUIDE-seq, Chip-seq, DISCOVER-seq, BLESS, etc.). These unbiased tools remove the chances of bias and the requirements for cell culture and transfections.

## 8. Conclusions and Future Prospects

Since the advent of CRISPR/Cas9 technology, many interdisciplinary researchers have worked to increase its efficiency and specificity. Rapid developments in bioinformatics tools have accelerated the quick application of CRISPR/Cas9, particularly due to the design of optimal and highly specific gRNA and post-genome-editing outcome analysis. To date, many computational tools have been designed for the analysis of off-target and on-target hits and their impacts. Many of these web gRNA design tools are publicly available for use. Still, there is a gap, that is, researchers are unable to understand the exact mechanisms of CRISPR/cas9 off-target effects and are unable to design a single web tool for designing gRNA with zero off-target effects. The development of a scoring-based model for off-target prediction depends on the DSB generation. Additionally, there are myriad types of tools for designing gRNAs and predicting off-target effects and impacts. Selecting the right tool for a specific experiment is very critical and depends on which species is being used to perform the CRISPR editing experiment and which tool and Cas nuclease enzyme work for that type of organism. Generally, for gRNA on-target activity, we suggest a scoring algorithm tool such as CRISPOR. Currently, before an experiment, InDelphi is the most advanced predictive tool to analyze off-target effects in vivo and in vitro. In a post-experiment analysis or outcome analysis, TIDE has outperformed others in finding off-target effects. The application of TIDE is limited due to the INDELS detection range of 30 to 50 bp, so larger nucleotide deletions cannot be detected. This limitation can be overcome by applying NGS off-target analysis, but NGS is expensive and time-consuming. Still, NGS is reliable for the better analysis of CRISPR/cas9 experiments.

To date, various types of base editors, prime editors, and epigenetic editors have been designed that can edit any gene without the generation of a DSB. Base editors, prime editors, and epigenetic editors have great future potential to treat genetic diseases through specific single-base mutations at specific sites. Without a DSB, prime editors can perform the desired insertions and deletions, as well as all 12 possible types of conversions. Prime editing has been adopted for many model organisms as well, mostly for food crops. In prime editing, all of the work is completed by pegRNA. Compared to the 5′ end of conventional single gRNA, pegRNA can be customized to work at the 3′ end. The 3′ end custom site is responsible for guide repair, the process of prime editing (PBS), and annealing of the nicked DNA strand. The GC content and RNA secondary structure affect the efficiency of pegRNA [80,81]. There are still large amounts of data required for generating off-target prediction tools and guide pegRNA design web tools. The majority of off-target prediction models depend on basic sequence features, and there is still room for improvement in understanding chromatin accessibility, thermodynamic features, and the exact gRNA and Cas9 cleavage process. To date, all empirical algorithms for designing gRNAs have been derived from large-scale gRNA data analysis on humans and the zebrafish model organism, but recent studies have revealed that off-target events vary from species to species: for example, the chances of off-target effects are low in plants as compared to animals [82,83]. Chromatin accessibility plays a very important role in gRNA activity and varies among species [84,85,86], so the comprehensive analysis of chromatin data across organisms could provide a new perspective on the development of the most efficient gRNA off-target prediction models to optimize CRISPR/Cas9 experiments. Recently, researchers from UC Barkley identified the anti-CRISPR protein (ACR11A4), which blocks the CRISPR/cas9 working mechanism. In their experiment, they found that delivering Cas9/gRN first and then the anti-CRISPR protein, ACR11A4, several hours later to human cell lines significantly reduced the CRISPR/Cas9 off-target effects. Researchers are currently using the anti-CRISPR strategy to reduce off-target effects in plants and animals [87].

Another important factor is the occurrence of genetic variance across species. The majority of designed off-target prediction models contain large-scale data on humans and do not have genetic variation data from other important species. As has been speculated, SNP mutations and variability also affect Cas9 cleavage efficiency [88]. Genetic variation has distributed PAMs across genomes. A few recently developed tools, such as CRISPROR and CCTop, incorporate genetic variation data. Recently, Yun et al. reported that different computational tools are recommended for different types of experiments [89]. In the future, developing a machine-learning-based model to incorporate sequence, structural, and epigenetic features, genetic variation data, and chromatin accessibility data would be the best tool to predict off-target as well as on-target activity.

## Figures and Tables

**Table 2:** Commonly used gRNA design tools.

Tool	Organism	Language	Cas Nucleases	Description	Database/ Web Server	Web Site (Accessed on 10 November 2022)	Ref.
CHOP CHOP	More than 100	Python	Cas9, Cas12, Cas13	Early web-based tool created by Harvard University to design gRNA based on matches and mismatches	Web server	http://crispor.tefor.net/	[36]
CRISPR RGNE tools	More than 100	Python	More than 20 Cas nucleases	Predicts multiple off-target and on-target effects based on the Cas-OFFinder model	Web server	http://www.rgenome.net/cas-designer/	
CRISTA	More than 100	Pearl and Python	Only Cas9	Machine learning (ML)-based tools to predict off-target and on-target effects simultaneously	Web server		[35]
GuideScan	Mouse and human	Python	Cpf1 and Cas9	Predicts off-target effects based on sequence and structural features	Web server	https://guidescan.com/	[37]
CRISPRDo	Human, mouse, zebrafish, and some worm species	Python	Cas9 and Cpf1	Predict off-target and on-target effects simultaneously	Database		[38]
sgRNACas9	Mouse	Pearl	spCas9	A web-based tool to predict off-target effects	Dataset		[39,40]
EupaDGT	Eukaryotic pathogen	Python	More than 10 Cas nucleases	Machine-learning-based tool to predict on- and off-target effects simultaneously	Web-based	http://grna.ctegd.uga.edu/	[41]
WU-CRISPR	Human and mouse	Pearl	SpCas9	Machine-learning-algorithm-based tool that can predict off-target effects by providing sequences between 20 and 30,000 bp	Web-based	http://crispr.wustl.edu/	[42]
CRISPR-P	49 plant species	Python	More than 14 Cas nucleases orthologs	Web-based off-target and on-target prediction tools for a wide range of plant species	Web-based	http://crispr.hzau.edu.cn/CRISPR2	[43]
CRISPRz	Zebrafish, human, and mouse	Python	spCas9	Trained on large datasets from zebrafish, humans, and mice to generate a gRNA dataset	Web-based	https://research.nhgri.nih.gov/CRISPRz/	[44]
PhytoCRISPR x	Wide range of plant species and especially phytoplankton	Pearl/bash	SpCas9. Cas12	Web-based tool to predict off-target effects	Dataset		[46]
CRISPRPOR	More than 100 species	Python	More than 30 Cas orthologs	Design gRNA dataset based on match/match in seed regions	Web tool	http://crispor.tefor.net/	[45]
Png Designer	6	Python	Cas9	A newly designed tool for generating guide RNA for base editing and prime editing	Web tool	https://www.crisprindelphi.design/	[47]

**Table 3:** Repair outcome prediction tools.

Model	Repair Prediction	Cell Lines	Remarks	Web Site (Accessed on 10 November 2022)	Ref.
FORECast	Can predict deletions as well as insertions, with 420 and 20 classes, respectively	iPSC, CHOHAP1, mESCs, K562, and RPE1	Created through multi-class logistic regression	https://partslab.sanger.ac.uk/FORECasT	[66]
CROTON	K562	Can predict the in-frameshift frequency with 1/2 bp	Created through CNN+ NAS	https://github.com/vli31/CROTON	[67]
InDelphi	HEK293, K562, HCT116, mESCs, and U20S	Can predict microhomology deletions (90 classes), non-microhomology deletions (59 classes), and 4 classes of 1 bp insertions	Generated through deep neural network and K-Nearest Neighbor	https://indelphi.giffordlab.mit.edu/about	[68]
Lindel	HEK293T	Can predict deletions (536 classes) and insertions (21 classes)	Generated through logistic regression	https://lindel.gs.washington.edu/Lindel	[69]
SPROUT	T cells	Can predict repairs such as INDELS	Gradient Boosting Decision Tree	https://zou-group.github.io/SPROUT	[70]
Apindel	K562	Can predict insertions (536 classes) and deletions (21 classes)	Glove + Positional Encoding		[71]

**Table 4:** Tools for post-experiment off-target analysis.

Tools	Analysis Basis	Input	Output	Supported Experiment	Supported Cas9 Nucleases	Ref.
CRISPResso2	NGS	FASTQ	Sequence alignment, NHEJ/HDR events	For CRISPR/Cas9, base editors	Cas9, Cpf1	[78]
CasAnalyzer	NGS	FASTQ	Sequence alignment, HDR/NHEJ events	CRISPR/Cas9	spCas9, StCas9, HFCas9, SaCas9, Cpf1, CjCas9	[77]
CRISPR-GA	NGS	FASTQ	INDEL frequency, recombination due to HRD events	Only Cas9	Cas9	[76]
TIDE/TIDER	Sanger	ABI	INDELS/HDR frequency	Only CRISPR	spCas9, SaCas9, FnCas9, AsCpf1, stCas9	[72]
ICE	Sanger	ABI	INDELS/HDR frequency	Only CRISPR/Cas9	Cas9, SaCas9	[79]

## Data Availability

Not applicable.
